# Exosomal noncoding RNAs as noninvasive biomarkers in bladder cancer: a diagnostic meta-analysis

**DOI:** 10.1007/s12094-023-03374-4

**Published:** 2024-01-16

**Authors:** Liming Zhao, Jun Li, Zhongguang Xue, Jinfeng Wang

**Affiliations:** Department of Nuclear Medicine, Linyi People’s Hospital, Shandong University, 27 Jiefang Road, Linyi, 276003 Shandong People’s Republic of China

**Keywords:** Exosome, NcRNAs, Bladder cancer, Noninvasive diagnosis, Meta-analysis

## Abstract

**Background:**

In view of discordance consisting in different reports, a meta-analysis was conducted to comprehensively evaluate the diagnostic efficacy of exosomal noncoding RNAs (ncRNAs) in blood and urine in the detection of bladder cancer.

**Methods:**

Eligible studies were acquired by systematic retrieval through PubMed, Cochrane Library, and Embase. The pooled diagnostic efficacy was appraised by reckoning the area under the summary receiver operating characteristic (SROC) curve. The latent sources of heterogeneity were probed by subgroup analyses and meta-regression. STATA 12.0, Meta-DiSc 1.4, and RevMan 5.3 were applied to carry out all statistical analyses and plots.

**Results:**

A total of 46 studies from 15 articles comprising 2622 controls and 3015 bladder cancer patients were included in our meta-analysis. Exosomal ncRNAs in blood and urine represented relatively satisfactory diagnostic efficacy in detecting bladder cancer, with a pooled sensitivity of 0.75, a specificity of 0.79, and an area under the SROC curve (AUC) of 0.84. Exosomal microRNAs (miRNAs) exhibited better diagnostic value with a pooled AUC of 0.91 than that of exosomal long noncoding RNAs (lncRNAs). To some extent, the heterogeneity among studies was induced by exosomal ncRNA types (miRNA or lncRNA), exosomal ncRNA profiling (single- or multiple-ncRNA), sample size, specimen types, and ethnicity.

**Conclusion:**

Exosomal ncRNAs in blood and urine may play a vital role in diagnosing bladder cancer as prospective noninvasive biomarkers; nonetheless, their clinical performance needs to be confirmed by further massive proactive researches.

**Supplementary Information:**

The online version contains supplementary material available at 10.1007/s12094-023-03374-4.

## Introduction

Bladder cancer is the 10th most commonly diagnosed cancer worldwide, with approximately 573,000 new cases and 213,000 deaths [1]. High mortality and frequent recurrence are the marked features of bladder cancer while it progresses into invasive stage [2]. The limitations of urine cytology (less sensitive for low-grade tumor) and cystoscopy (invasive and hard to detect carcinoma *in situ*) have been major hindrances to their clinical practice as diagnostic and surveillance strategies for bladder cancer [3]. Given this, there is an urgent need for investigating original noninvasive biomarkers with high diagnostic accuracy for bladder cancer.

Exosomes, as a “bridge” between cells, may be engaged in tumorigenesis and tumor development by delivering abundant elements including ncRNAs (consisting of miRNA, lncRNA, circular RNA, small interfering RNA, small nuclear RNA, small nucleolar RNA, and PIWI-interacting RNA) [4]. Lately, plenty of studies have confirmed the stability of ncRNAs derived from exosomes in body fluids and the feasibility of them as noninvasive biomarkers for diagnosing bladder cancer [5–19]. In light of discrepancies existing among these studies with regard to the reliability of exosomal ncRNAs for the detection of bladder cancer, we implemented this meta-analysis to synthetically clarify the diagnostic significance of exosomal ncRNAs for bladder cancer based on published studies.

## Materials and methods

We carried out and reported this research abiding by the Preferred Reporting Items for Systematic Reviews and Meta-Analysis for Diagnostic Test Accuracy (PRISMA-DTA) guidelines [20], and the corresponding checklists are displayed in the Supplemental File. The research has been registered in the PROSPERO database with the number CRD42023484273.

### Eligibility criteria

The following PICOS criteria were adopted to judge study eligibility: (1) participants: studies evaluating untreated bladder cancer participants (regardless of gender, age, and race); (2) index tests: exosomal ncRNAs in blood and urine; (3) comparative test: comparative tests would not be mandatory provided the study assessed the diagnostic efficiency of exosomal ncRNAs for bladder cancer; (4) outcomes: pooled sensitivity, specificity, positive likelihood ratio (PLR), negative likelihood ratio (NLR), diagnostic odds ratio (DOR), and AUC; (5) study type: studies appraising the diagnostic property of exosomal ncRNAs for bladder cancer (regardless of the type of study); and (6) reference standard: histopathology.

### Bibliographic search

Eligible articles regarding the diagnostic importance of exosomal ncRNAs for bladder cancer were obtained through systematic document retrieval of PubMed, Cochrane Library, and Embase for researches published in English till September 20, 2023. The following retrieval strategy was carried out: (“exosome” OR “exosomal”) AND (“noncoding RNA” OR “long noncoding rna” OR “lncrna” OR “microRNA” OR “miRNA” OR “miR” OR “CircRNA” OR “small interfering RNA” OR “small nuclear RNA” OR “small nucleolar RNA” OR “PIWI-interacting RNA”) AND (“urinary bladder neoplasms” OR “bladder cancer” OR “bladder tumor” OR “transitional cell carcinoma of bladder” OR “bladder carcinoma” OR “urinary tract transitional cell carcinoma”) AND (“blood” OR “serum” OR “plasma” OR “urine” OR “urinary”) AND (“diagnosis” OR “sensitivity and specificity” OR “ROC curve”). Extra articles were acquired by manually retrieving references list of all the involved publications.

### Inclusion and exclusion criteria

Two reviewers independently appraised the qualified articles, yet consensus was reached by multilateral discussion with a third reviewer if any dispute arose. The inclusion criteria must be met as follows: (1) the diagnostic performance of exosomal ncRNAs in blood and urine for bladder cancer was explored; (2) histopathology was adopted to confirm the diagnosis of patients with bladder cancer; and (3) sufficient data could be extracted for rebuilding two-by-two tables consisting of true positive (TP), false positive (FP), true negative (TN), and false negative (FN). The exclusion criteria were: (1) studies unrelated to the diagnostic value of exosomal ncRNAs for bladder cancer; (2) studies with low quality, deficient data, or replicated data; and (3) meta-analysis articles, meeting reports, reviews, case reports, or seminar articles.

### Data extraction

Standardized forms were exploited by two reviewers to independently extract the following data from all eligible studies: (1) basic features of studies, comprising first author, publication year, country of studies, publication journal, ethnicity, sample size, cancer type, tumor stage, tumor grade, mean age, gender ratio, specimen type, ncRNA profiling, reference gene, assay methods and (2) diagnostic efficiency, consisting of TP, FP, FN, TN, sensitivity, specificity, and AUC. We used Kappa score to evaluate inter-rater agreement based on the extracted data above.

### Quality evaluation

Two reviewers systematically evaluated and independently rated each included study in accordance with the revised Quality Assessment of Diagnostic Accuracy Studies 2 (QUADAS-2) criteria [21] recommended by *Cochrane Handbook for Systematic Reviews of Diagnostic Test Accuracy* [22] as we described in a previous article [23]. Any disagreement was settled by discussing with a third reviewer to reach a consensus.

### Statistical analysis

The *I*^2^ statistic and *Q* test were utilized to evaluate significant heterogeneity (*I*^2^ value ≥ 50% or *P* value < 0.10 for the *Q* test, then employing the random-effects model) among the included studies [24]. Furthermore, the potential sources of heterogeneity were clarified by meta-regression and subgroup analyses. Pooled sensitivity, specificity, PLR, NLR, and DOR of all the included studies were figured out by applying a bivariate meta-analysis model. The sensitivity and specificity of each included study were adopted to plot the SROC curve, with AUC demonstrating pooled diagnostic value. The post-diagnostic effect after pooled analysis was appraised by the Fagan’s nomogram. The publication bias was estimated by Deek’s funnel plot asymmetry test, with *P* < 0.10 indicating significant difference [25]. All analyses and plots were performed by the STATA 12.0 (StataCorp LP, College Station, TX, USA), Meta-DiSc 1.4 (Ramony Cajal Hospital, Madrid, Spain), and RevMan 5.3 (Nordic Cochrane Centre, Copenhagen, Denmark).

## Results

### Document retrieval

**Fig. 1:**
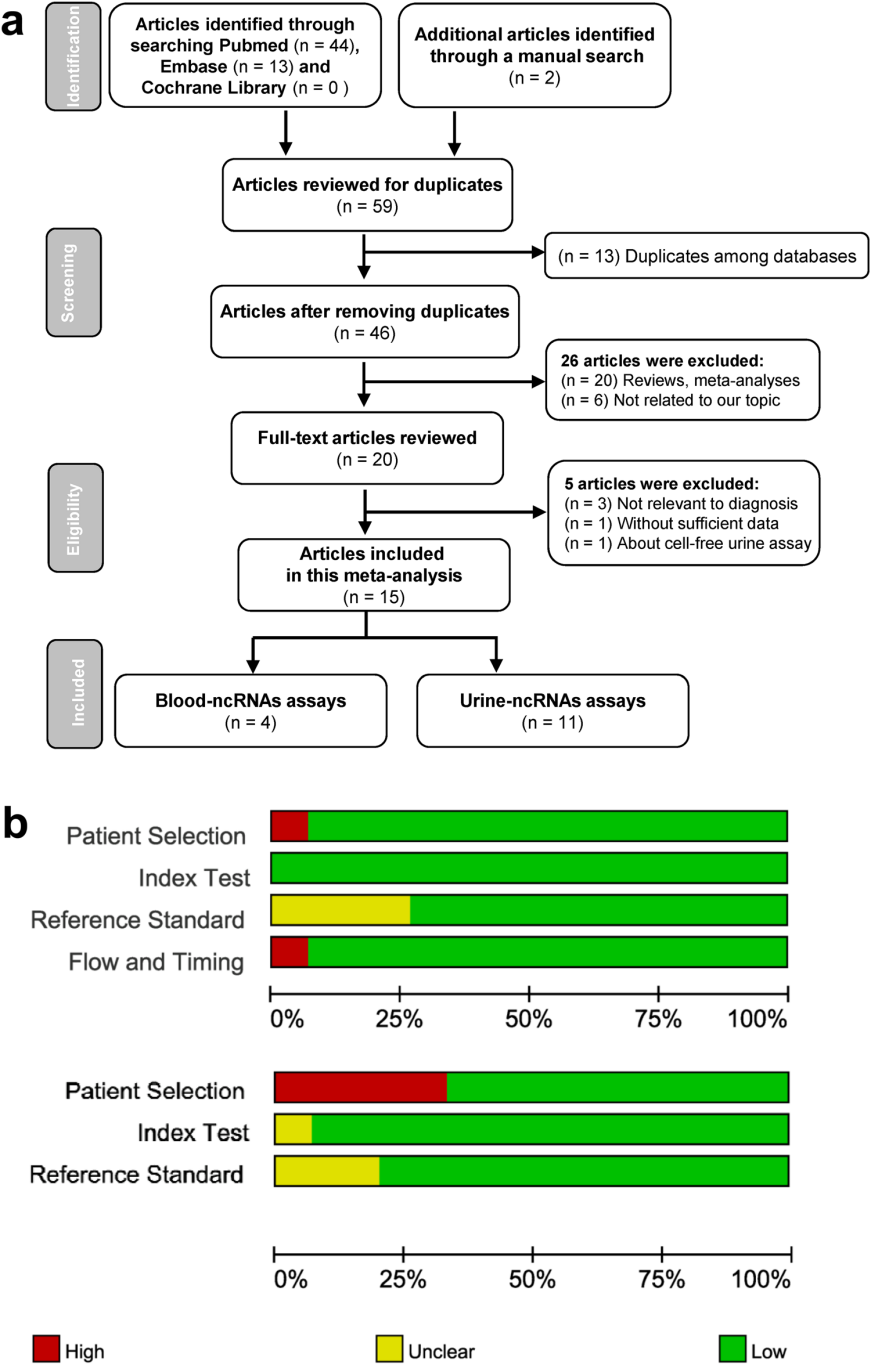
Flow chart of publications selection process (**a**) and quality assessment of included publications using QUADAS-2 criteria (**b**)

The process of bibliographic search is demonstrated in Fig. 1a. By initial database search, a total of 59 potentially relevant articles were acquired, of which 13 duplicates were taken out. Twenty meta-analysis and reviews and six articles uncorrelated to our theme were further removed through abstract appraisal. Next, 20 articles were retained for the full-text review, of which 5 articles (3 articles irrelevant to diagnosis, 1 article without adequate data, and 1 article about cell-free urine assay) were eliminated. Ultimately, 15 eligible articles were incorporated in this meta-analysis, consisting of 4 articles involving blood-based exosomal ncRNAs [5, 7, 9, 10] and 11 articles about urine-based ones [6, 8, 11–19].

### General features of included studies

Table 1 indicates the fundamental characteristics of the included publications. A total of 46 studies from 15 articles published from 2017 to 2023 were adopted in our meta-analysis, comprising 2622 controls and 3015 bladder cancer patients. Thirty-one studies examined Asian participants and 15 studies exploited Caucasian participants. Thirty-six studies referred to diagnostic implication of exosomal ncRNAs in both muscle invasive bladder cancer (tumor stage T2-T4) and nonmuscle invasive bladder cancer (tumor stage Tis, Ta-T1). Forty-five studies involved both low- and high-grade bladder cancer. Six studies investigated exosomal miRNAs, while forty studies concerned lncRNAs derived from exosomes. Only 12 studies focused on exosomal multiple-ncRNA assay in bladder cancer detection, while 34 studies investigated single-ncRNA assay, of which 7 studies probed into the diagnostic value of exosomal single UCA1 in bladder cancer and 6 studies explored single MALAT1. Quantitative real-time reverse transcription-polymerase chain reaction (qRT-PCR) was employed to determine the expression level of exosomal ncRNAs in all the included studies. All the included studies normalized the ncRNA concentration to reference gene. Five studies concerned blood-based specimen (four studies based on serum and one based on plasm), and the other 41 studies exploited urine-based specimen. The Kappa score for data extraction was 1.0 (95% CI: 0.9–1.0), manifesting an ideal level of inter-rater agreement. As depicted in Fig. 1b, the majority of studies involved in this meta-analysis fulfilled at least four items in QUADAS-2 tool, manifesting good overall quality of the included studies.

**Table 1:** Fundamental characteristics of the 46 studies included in the meta-analysis

Included studies	Country	Ethnicity	Study design	Case/control			Cancer type	Tumor stage	Tumor grade	NcRNA profiling	Reference gene	Specimen	QUADAS
				Number	Mean age	Male ratio							
Xue M, 2017	China	Asian	Case–control	30/30	NA/NA	0.73/NA	BCa	16T1 + 14T2-T4	9Low + 21High	UCA1	GAPDH	Serum	5
Zhan Y, 2018	China	Asian	Case–control	104/104	65/53	0.79/0.76	BCa	61Ta-T1 + 43T2-T4	46Low + 58High	MALAT1	GAPDH	Urine	7
				104/104	65/53	0.79/0.76	BCa	61Ta-T1 + 43T2-T4	46Low + 58High	PCAT-1	GAPDH	Urine	
				104/104	65/53	0.79/0.76	BCa	61Ta-T1 + 43T2-T4	46Low + 58High	SPRY4-IT1	GAPDH	Urine	
				80/80	65/53	0.81/0.75	BCa	50Ta-T1 + 30T2-T4	39Low + 41High	MALAT1	GAPDH	Urine	
				80/80	65/53	0.81/0.75	BCa	50Ta-T1 + 30T2-T4	39Low + 41High	PCAT-1	GAPDH	Urine	
				80/80	65/53	0.81/0.75	BCa	50Ta-T1 + 30T2-T4	39Low + 41High	SPRY4-IT1	GAPDH	Urine	
				104/104	65/53	0.79/0.76	BCa	61Ta-T1 + 43T2-T4	46Low + 58High	3 lncRNAs^a^	GAPDH	Urine	
				80/80	65/53	0.81/0.75	BCa	50Ta-T1 + 30T2-T4	39Low + 41High	3 lncRNAs^a^	GAPDH	Urine	
Zheng R, 2018	China	Asian	Case–control	50/60	67/66	0.72/0.73	BCa	41Ta-T1 + 9T2-T4	21G1 + 14G2 + 15G3	PTENP1	GAPDH	Plasm	5
Yazarlou F, 2018	Iran	Caucasian	Case–control	59/49	61/64	1.00/1.00	TCC	NA	20Low + 28High + 11NA	UCA1-201	5S rRNA	Urine	3
				59/49	61/64	1.00/1.00	TCC	NA	20Low + 28High + 11NA	UCA1-203	5S rRNA	Urine	
				59/49	61/64	1.00/1.00	TCC	NA	20Low + 28High + 11NA	MALAT1	5S rRNA	Urine	
				59/49	61/64	1.00/1.00	TCC	NA	20Low + 28High + 11NA	4 lncRNAs^b^	5S rRNA	Urine	
				59/24	61/68	1.00/1.00	TCC	NA	20Low + 28High + 11NA	UCA1-201	5S rRNA	Urine	
				59/24	61/68	1.00/1.00	TCC	NA	20Low + 28High + 11NA	UCA1-203	5S rRNA	Urine	
				59/24	61/68	1.00/1.00	TCC	NA	20Low + 28High + 11NA	MALAT1	5S rRNA	Urine	
				59/24	61/68	1.00/1.00	TCC	NA	20Low + 28High + 11NA	LINC00355	5S rRNA	Urine	
				59/24	61/68	1.00/1.00	TCC	NA	20Low + 28High + 11NA	4 lncRNAs^b^	5S rRNA	Urine	
Wang J, 2018	China	Asian	Case–control	52/104	65/NA	0.75/NA	BCa	29Ta-T1 + 23T2-T4	24Low + 28High	H19	GAPDH	Serum	4
Zhang S, 2019	China	Asian	Case–control	100/100	60/60	0.81/0.73	BCa	56Ta-T1 + 44T2-T4	48Low + 52High	3 lncRNAs^c^	GAPDH	Serum	7
				160/160	60/60	0.78/0.66	BCa	84Ta-T1 + 76T2-T4	66Low + 94High	3 lncRNAs^c^	GAPDH	Serum	
Abbastabar M, 2020	Iran	Caucasian	Case–control	30/10	55.8/57.4	NA/NA	BCa	20T1 + 10T2	13Low + 17High	ANRIL	5 s rRNA	Urine	6
				30/10	55.8/57.4	NA/NA	BCa	20T1 + 10T2	13Low + 17High	PCAT-1	5 s rRNA	Urine	
Huang H, 2021	China	Asian	Case–control	80/80	64.8/47.4	0.83/0.70	BCa	28Ta + 36T1 + 16T2-T4	35Low + 45High	MIR205HG	GAPDH	Urine	7
				80/80	64.8/47.4	0.83/0.70	BCa	28Ta + 36T1 + 16T2-T4	35Low + 45High	GAS5	GAPDH	Urine	
				80/80	64.8/47.4	0.83/0.70	BCa	28Ta + 36T1 + 16T2-T4	35Low + 45High	2 lncRNAs^d^	GAPDH	Urine	
Chen C, 2021	China	Asian	Case–control	242/166	65/NA	0.75/NA	BCa	79T1 + 163T2-T4	65Low + 177High	ELNAT1	GAPDH	Urine	7
Sarfi M, 2021	Iran	Caucasian	Case–control	30/10	62.7/57.4	NA/NA	BCa	20Ta-T1 + 10T2	13Low + 17High	TUG-1	5 s rRNA	Urine	6
El-Shal AS, 2021	Egypt	Caucasian	Case–control	51/49	59.5/58	0.82/0.84	BCa	22Ta-T1 + 29T2-T4	42Low + 9High	miR-96-5p	SNORD68	Urine	7
				51/49	59.5/58	0.82/0.84	BCa	22Ta-T1 + 29T2-T4	42Low + 9High	miR-183-5p	SNORD68	Urine	
				51/49	59.5/58	0.82/0.84	BCa	22Ta-T1 + 29T2-T4	42Low + 9High	2 miRNAs^e^	SNORD68	Urine	
Lin H, 2021	China	Asian	Case–control	53/51	65/62	0.75/0.85	BCa	32Ta-T1 + 21T2-T4	22Low + 31High	miR-93-5p	U6 snRNA	Urine	7
				53/51	65/62	0.75/0.85	BCa	32Ta-T1 + 21T2-T4	22Low + 31High	miR-516a-5p	U6 snRNA	Urine	
				53/51	65/62	0.75/0.85	BCa	32Ta-T1 + 21T2-T4	22Low + 31High	2 miRNAs^f^	U6 snRNA	Urine	
Chen C, 2022	China	Asian	Case–control	89/63	NA/NA	NA/NA	BCa	NA	NA	TERC	18S	Urine	6
Qiu T, 2022	China	Asian	Case–control	22/20	62/63.5	0.91/0.95	BCa	19Ta-T1 + 3T2-T4	15Low + 7High	RMRP	GAPDH	Urine	7
				22/20	62/63.5	0.91/0.95	BCa	19Ta-T1 + 3T2-T4	15Low + 7High	UCA1	GAPDH	Urine	
				22/20	62/63.5	0.91/0.95	BCa	19Ta-T1 + 3T2-T4	15Low + 7High	MALAT1	GAPDH	Urine	
				22/20	62/63.5	0.91/0.95	BCa	19Ta-T1 + 3T2-T4	15Low + 7High	3 lncRNAs^g^	GAPDH	Urine	
				33/23	65/65	0.85/0.74	BCa	22Ta-T1 + 11T2-T4	14Low + 19High	3 lncRNAs^g^	GAPDH	Urine	
				55/43	NA/NA	0.87/0.84	BCa	41Ta-T1 + 14T2-T4	29Low + 26High	3 lncRNAs^g^	GAPDH	Urine	
				55/43	NA/NA	0.87/0.84	BCa	41Ta-T1 + 14T2-T4	29Low + 26High	RMRP	GAPDH	Urine	
				55/43	NA/NA	0.87/0.84	BCa	41Ta-T1 + 14T2-T4	29Low + 26High	UCA1	GAPDH	Urine	
				55/43	NA/NA	0.87/0.84	BCa	41Ta-T1 + 14T2-T4	29Low + 26High	MALAT1	GAPDH	Urine	
Liu C, 2023	China	Asian	Case–control	42/42	60/NA	0.79/NA	BCa	18Ta-T1 + 24T2-T4	13Low + 29High	SNHG16	18S	Urine	6

^a^lnc-MALAT1, PCAT-1, SPRY4-IT1

^b^lnc-UCA1-201, UCA1-203, MALAT1, LINC00355

^c^lnc-PCAT-1, UBC1, SNHG16

^d^lnc-MIR205HG, GAS5

^e^miR-96-5p,-183-p

^f^miR-93-5p, -516a-5p

^g^lnc-RMRP, UCA1, MALAT1

*NA* not available, *TCC* transitional cell carcinoma

### Diagnostic performance of exosomal ncRNAs for bladder cancer

**Fig. 2:**
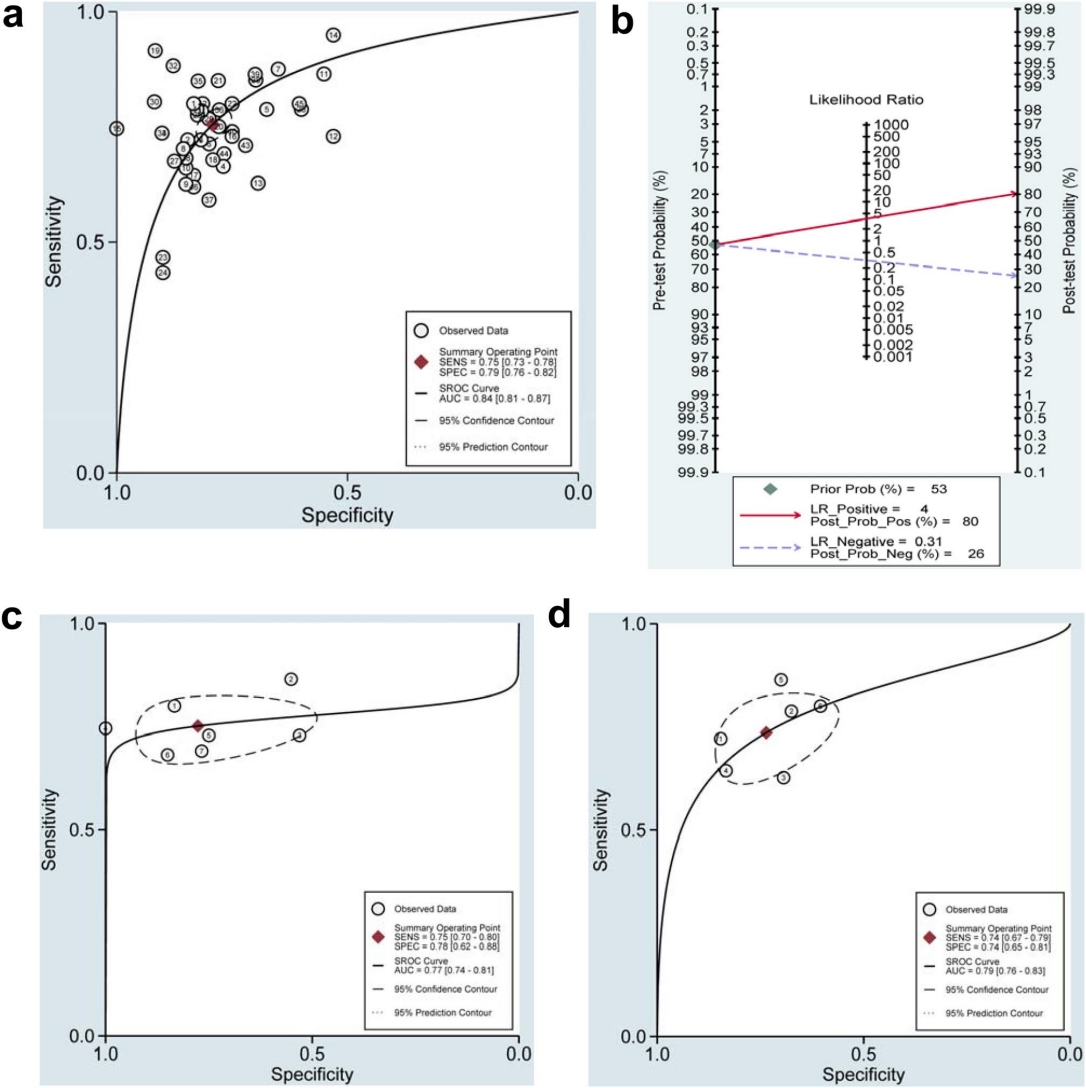
Diagnostic efficacy of exosomal ncRNAs for bladder cancer (**a**) SROC curve with pooled estimates of sensitivity, specificity, and AUC of overall studies (**b**) Fagan’s nomogram for appraisal of post-test probabilities based on pooled estimates of PLR and NLR of overall studies (**c**) SROC curves based on the diagnostic studies of UCA1 and (**d**) SROC curves based on the diagnostic studies of MALAT1

**Fig. 3:**
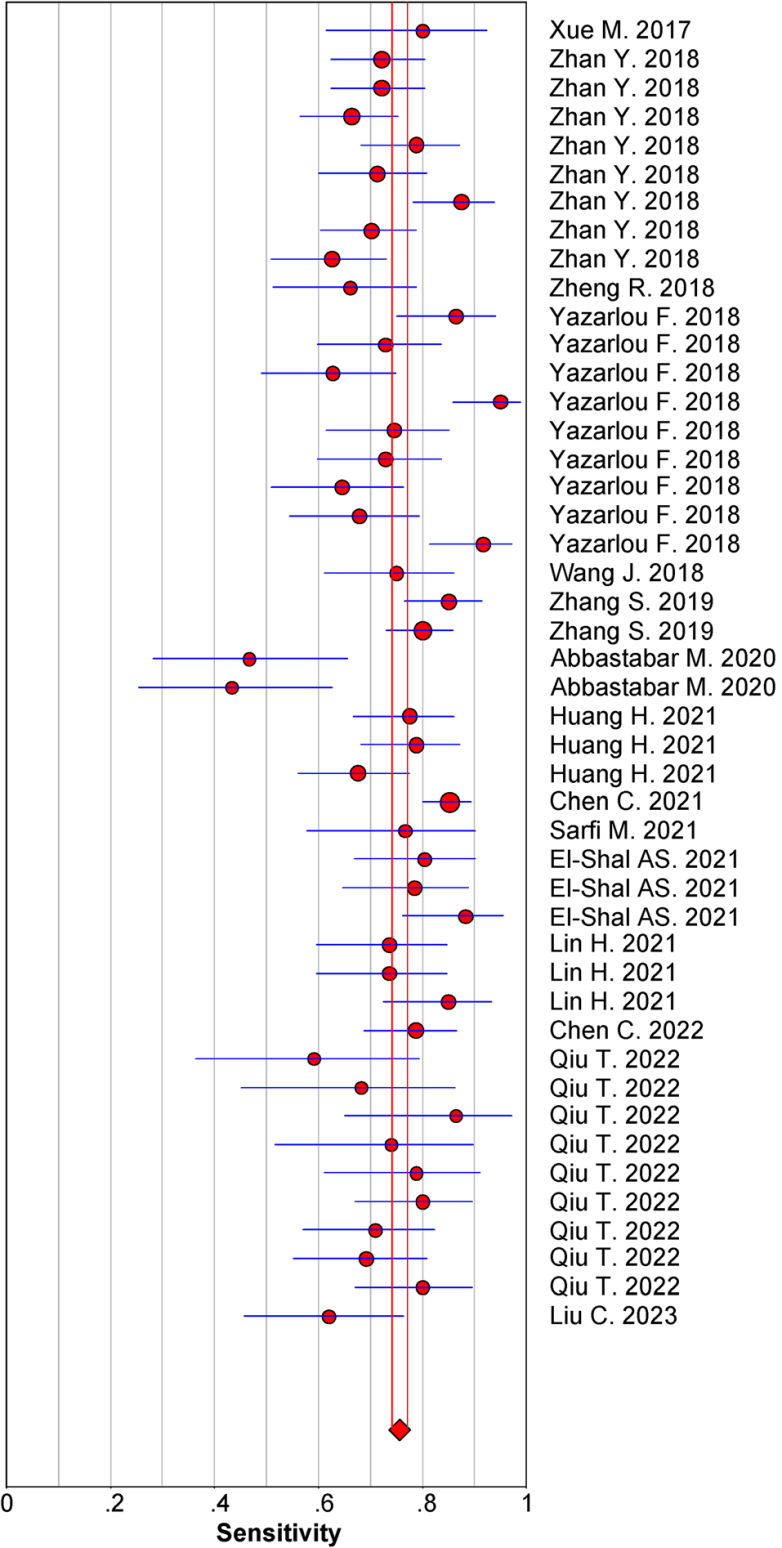
Forest plot displaying the weight and sensitivity of overall studies (size of red dot represents how much that particular study contributes to the overall statistic; red diamond indicates pooled sensitivity; blue horizontal line denotes 95% CI of sensitivity)

Given that significant heterogeneity among studies observed in sensitivity (*I*^2^ = 66.64%) and specificity (*I*^2^ = 68.34%) (*P* < 0.01), the random-effects model was adopted accordingly. As shown in Table 2, the pooled parameters reckoned from all the 46 studies were as follows: sensitivity, 0.75 (95% CI: 0.73–0.78); specificity, 0.79 (95% CI: 0.76–0.82); PLR, 3.6 (95% CI: 3.2–4.1); NLR, 0.31 (95% CI: 0.28–0.34); DOR, 12 (95% CI: 10–14); and AUC, 0.84 (95% CI: 0.81–0.87) (Fig. 2a), implying that exosomal ncRNAs in blood and urine may be a qualified diagnostic indicator for bladder cancer with moderate accuracy. As demonstrated in the Fagan’s plot (Fig. 2b), the pre-test probability was 53%, and the post-test probability of bladder cancer for a positive test result was 80%, while that for a negative test result was 26%, revealing that both the post-test probabilities and likelihood ratios were moderate. The PLR of 3.6 displayed that a person with bladder cancer is 3.6 times more likely to have a positive test result than a healthy person. Moreover, the DOR value was 12 (95% CI: 10–14), which signified that exosomal ncRNAs in blood and urine can be utilized to differentiate bladder cancer patients from controls. In addition, exosomal single MALAT1 presented relatively good diagnostic accuracy with a pooled AUC of 0.79 than that of UCA1 with a pooled AUC of 0.77 (Table 2 and Fig. 2c, d). However, there was no significant difference between the pooled AUC of the two exosomal ncRNAs (*Z* value = 0.30, *P* for *Z* test > 0.05). Figure 3 demonstrates the weight and sensitivity of each study with a pooled sensitivity of 0.75 (95% CI: 0.73–0.78) (*P* < 0.001).

**Table 2:** Summary estimates of diagnostic efficacy for exosomal ncRNAs profiling in bladder cancer detection

Analysis	No. of studies	SEN (95% CI)	SPE (95% CI)	PLR (95% CI)	NLR (95% CI)	DOR (95% CI)	AUC (95% CI)
Overall	46	0.75 (0.73–0.78)	0.79 (0.76–0.82)	3.6 (3.2–4.1)	0.31 (0.28–0.34)	12 (10–14)	0.84 (0.81–0.87)
UCA1	7	0.75 (0.70–0.80)	0.78 (0.62–0.88)	3.4 (1.9–5.9)	0.32 (0.26–0.40)	10 (5–22)	0.77 (0.74–0.81)
MALAT1	6	0.74 (0.67–0.79)	0.74 (0.65–0.81)	2.8 (2.1–3.7)	0.36 (0.29–0.44)	8 (5–12)	0.79 (0.76–0.83)
Exosomal ncRNA types							
MiRNA	6	0.80 (0.75–0.84)	0.87 (0.83–0.91)	6.3 (4.6–8.6)	0.23 (0.18–0.29)	27 (18–43)	0.91 (0.88–0.93)
LncRNA	40	0.75 (0.72–0.78)	0.78 (0.74–0.81)	3.4 (3.0–3.8)	0.32 (0.29–0.36)	10 (9–12)	0.83 (0.79–0.86)
Exosomal ncRNA profiling							
Single-ncRNA assay	34	0.74 (0.70–0.76)	0.79 (0.75–0.82)	3.4 (2.9–4.0)	0.34 (0.30–0.37)	10 (8–13)	0.82 (0.78–0.85)
Multiple-ncRNA assay	12	0.81 (0.74–0.86)	0.80 (0.75–0.85)	4.1 (3.3–5.1)	0.24 (0.18–0.31)	17 (13–24)	0.87 (0.84–0.90)
Specimen types							
Blood based	5	0.78 (0.72–0.83)	0.79 (0.74–0.83)	3.6 (3.0–4.4)	0.28 (0.22–0.36)	13 (9–19)	0.84 (0.81–0.87)
Urine based	41	0.75 (0.72–0.78)	0.79 (0.76–0.82)	3.6 (3.1–4.2)	0.31 (0.28–0.35)	12 (9–14)	0.84 (0.80–0.87)
Ethnicity							
Caucasian based	15	0.76 (0.68–0.83)	0.81 (0.72–0.88)	4.0 (2.6–6.0)	0.30 (0.22–0.40)	13 (8–24)	0.85 (0.82–0.88)
Asian based	31	0.75 (0.73–0.78)	0.79 (0.76–0.82)	3.6 (3.2–4.0)	0.31 (0.28–0.34)	14 (8–24)	0.84 (0.80–0.87)

*CI* confidence interval, *SEN* sensitivity, *SPE* specificity, *PLR* positive likelihood ratio, *NLR* negative likelihood ratio, *DOR* diagnostic odds ratio, *AUC* area under the curve

### Subgroup analysis

Subgroup analyses based on exosomal ncRNA types (miRNA or lncRNA), exosomal ncRNA profiling (single- or multiple-ncRNA), specimen types, and ethnicity were conducted separately, and the pooled results for diagnostic efficacy in different subgroups are displayed in Table 2. Exosomal miRNAs manifested significantly better diagnostic accuracy with a pooled AUC of 0.91 than that of exosomal lncRNAs (a pooled AUC of 0.83) (*Z* value = 3.87, *P* for *Z* test < 0.01). Exosomal multiple-ncRNA assays gave rise to superior pooled AUC of 0.87 to that of single ones (a pooled AUC of 0.82) (*Z* value = 3.37, *P* for *Z* test < 0.01). Urine-based assays and blood-based ones exhibited the same pooled AUC of 0.84 (*Z* value = 0.98, *P* for *Z* test > 0.05). Furthermore, the diagnostic property of Asian- versus Caucasian-based exosomal ncRNA assays was 0.84 versus 0.85 for pooled AUC (*Z* value = 0.45, *P* for *Z* test > 0.05).

### Meta-regression and publication bias

**Fig. 4:**
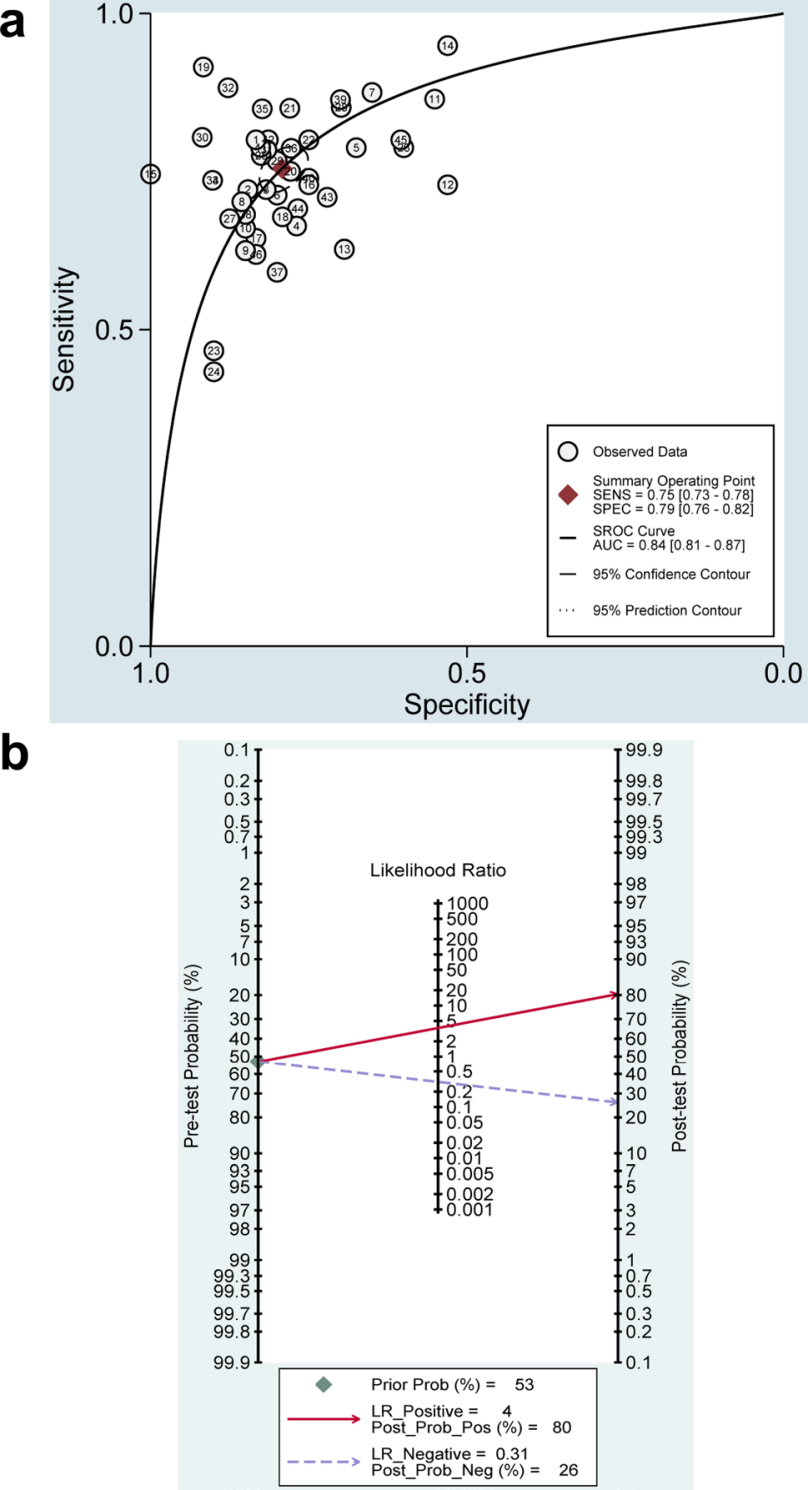
Meta-regression and publication bias based on overall studies (**a**) Forest plots of multivariable meta-regression analyses for sensitivity and specificity (vertical lines represent pooled estimates of sensitivity and specificity) (**b**) Deek’s funnel plot asymmetry test

The potential sources of the heterogeneity were further investigated by meta-regression analysis. Sample size, exosomal ncRNA types, exosomal ncRNA profiling, specimen types, and ethnicity were probably the main sources of heterogeneity for exosomal ncRNA assays in bladder cancer as depicted in Fig. 4a. As presented in Fig. 4b, the publication bias was assessed through Deek’s funnel plot asymmetry test, and there was no significant publication bias in our meta-analysis (*P* = 0.65).

## Discussion

Despite urine cytology and cystoscopy currently being the most generally utilized tools for diagnosing and surveilling bladder cancer, their clinical manifestations were unsatisfactory in some ways. It has been reported that ncRNAs (including microRNA, long noncoding RNA, and circular RNA) are sorted and packaged into exosomes selectively and transferred into recipient cells to regulate their function in bladder cancer [26]. At present, study on the potential of exosomal ncRNAs as biomarkers for bladder cancer has become a hot research topic [5–19]. This meta-analysis was designed to comprehensively expound the diagnostic significance of exosomal ncRNAs in detecting bladder cancer due to the existence of inconformity among these related studies.

Our report is, as yet, among the few evidence-based meta-analysis investigating the diagnostic performance of exosomal ncRNAs in discriminating bladder cancer with a pooled AUC of 0.84 (pooled sensitivity = 75%; pooled specificity = 79%), verifying the potential diagnostic efficacy of exosome-derived ncRNAs as noninvasive biomarkers. Exosomes, as lipid membrane-bound satchels with a multivesicular endosomal origin, are secreted by both normal and neoplastic cells and hence protect the contained soluble materials (such as nucleic acids, proteins, and lipids) from degradation in body fluid [27, 28]. Besides, the DOR was 12 (95% CI: 10–14), implying that exosomal ncRNA test-positive patients have 12-times higher chance of bladder cancer compared to controls.

Now that there were seven studies regarding the diagnostic implication of exosomal UCA1 and six studies concerning single MALAT1 in bladder cancer, and thus independent meta-analyses of the above two exosomal lncRNAs were carried out. Both single-exosomal UCA1 and single-exosomal MALAT1 exhibited relatively satisfactory diagnostic accuracy with an AUC of 0.77 and 0.79, respectively, manifesting their potential as noninvasive diagnostic biomarkers for bladder cancer. Nevertheless, no statistical difference was found between their pooled AUC.

Next, the potential sources of heterogeneity were investigated by subgroup analyses and meta-regression analysis. Our results displayed that exosomal ncRNA types (miRNA or lncRNA), exosomal ncRNA profiling (single- or multiple-ncRNA), sample size, specimen types, and ethnicity seemed to be the major sources of heterogeneity for exosomal ncRNA assays in bladder cancer because they had significant influence on the pooled sensitivity and specificity. First, miRNAs originating from exosomes exhibited higher diagnostic accuracy with a pooled AUC of 0.91 than that of exosome-derived lncRNAs (a pooled AUC of 0.83). Whereas, the results should be approached with prudence as only six included studies were regarding exosomal miRNAs. Furthermore, exosomal multiple-lncRNA assays generated a pooled AUC of 0.87, while single ones gave rise to a pooled AUC of 0.82, which demonstrated the superiority of exploiting panels of exosomal ncRNAs to get a full picture. As depicted in Table 1, exosomal lnc-MALAT1, PCAT-1, and SPRY4-IT1 from urine [6]; exosomal lnc-UCA1-201, UCA1-203, MALAT1, and LINC00355 from urine [8]; exosomal lnc-PCAT-1, UBC1, and SNHG16 from serum [10]; exosomal lnc-MIR205HG and GAS5 from urine [12]; exosomal miR-96-5p and miR-183-5p from urine [15]; exosomal miR-93-5p and miR-516a-5p from urine [16]; and exosomal lnc-RMRP, UCA1, and MALAT1 from urine [18] all manifested satisfying diagnostic value for bladder cancer. The potential molecular mechanism about the limitation of exosomal single-ncRNA biomarker maybe that aberrant levels of single ncRNA might be related to various types of cancers [29]. What is more, the development of cancer may be resulted by intricate multi-stage process of genomic and epigenetic abnormalities; hence, it also should be targeted by multiple-exosomal ncRNAs. Moreover, there seemed no clear difference between the diagnostic value of urine-based assays and blood-based ones, both with a pooled AUC of 0.84. However, it is advisable to treat the results with caution since there were merely five included studies involving blood-based assays. At last, ethnicity might be another feasible source of heterogeneity, with a pooled AUC of 0.85 for Caucasian-based exosomal ncRNA assays and 0.84 for Asian-based ones, which could be ascribed to different living environments and genetic elements between the two races. A large-scale survey should be carried out to elucidate whether this ethnicity-related variance truly exists.

In a meta-analysis from 2021, Su et al. appraised the diagnostic significance of exosome-derived lncRNAs for bladder cancer based on 23 studies from 10 articles (6 articles in urine and 4 articles in blood) including 1883 bladder cancer patients and 1721 controls and displayed that pooled sensitivity, specificity, and AUC of overall exosomal lncRNAs were 0.74, 0.76, and 0.83, respectively [30]. Whereas our report was designed to evaluate the diagnostic implication of exosomal ncRNAs (comprising exosomal miRNAs and lncRNAs) from urine, serum, and plasma, which differed from Su et al.’s. What is more, they did not carry out subgroup analyses based on sample size and ethnicity, which was less extensive than our report. In addition, our report demonstrated that exosomal miRNAs exhibited better diagnostic value with a pooled AUC of 0.91 than exosomal lncRNAs. Besides, we also testified that exosomal multiple-ncRNA assays gave rise to superior diagnostic efficacy to that of single ones. Lastly, we evaluated the diagnostic efficiency of the two most often investigated exosomal ncRNAs (MALAT1 and UCA1) and found no significant difference between them. The above aspects indicated that our report was novel and more comprehensive.

We did our utmost to conduct an accurate analysis; however, our report was imperfect. First, although we did not limit the type of study to be searched, eligible studies finally enrolled in this meta-analysis were all case–control ones, so patient selection was the major risk of bias across the enrolled documents. Since shortcomings in the design and conduct of test accuracy studies can lead to biased estimates of test accuracy, Cochrane recommends using the QUADAS-2 tool to evaluate the risk of bias and applicability of test accuracy studies [22]. As displayed in Fig. 1b, the majority of studies included in our meta-analysis fulfilled at least four items in the QUADAS-2 tool, demonstrating good overall quality. Besides, it is crucial to exploit panels of exosomal ncRNAs capable of discerning cancer from other diseases with similar symptoms. Nevertheless, most of the studies included in this meta-analysis only attempted to discriminate bladder cancer patients from healthy controls, not concerning patients with diseases of similar symptoms to bladder cancer. In addition, clinical practice of exosomal ncRNAs is sharply constrained because of their low abundance in body fluids and short of acknowledged endogenous reference genes. Accordingly, a standardized procedure should be established and preferably observed across all studies to minimize procedure-based bias. Moreover, in view of significant heterogeneity existing in our analysis owing to sample size, exosomal ncRNA types, exosomal ncRNA profiling, specimen types, and ethnicity, the results would inevitably be affected. Nevertheless, just as *Cochrane Handbook for Systematic Reviews of Diagnostic Test Accuracy* [22] states: “Heterogeneity is to be expected in meta-analyses of diagnostic test accuracy. A consequence of this is that meta-analyses of test accuracy studies tend to focus on computing average rather than typical effects. In systematic reviews of interventions, it is sometimes noted that the estimates of the effect of the intervention in the different studies are very similar, the differences between them being small enough to be explicable by chance. In systematic reviews of test accuracy, large differences are commonly noted between studies, too big to be explained by chance, indicating that actual test accuracy varies between studies: there is heterogeneity in test accuracy. Random-effects meta-analysis methods are recommended when effects are heterogeneous.” Furthermore, there has been no published data involving African populations. Finally, although some studies supplied information of tumor grade and stage and some even provided cutoff values of qRT-PCR and reference genes, subgroup analyses on the basis of these parameters were limited due to lack of data published.

## Conclusions

In this study, we reviewed the diagnostic efficacy of exosomal ncRNAs in blood and urine in the detection of bladder cancer. Nonetheless, their clinical performance needs to be confirmed by further massive proactive researches.

### Supplementary Information

Below is the link to the electronic supplementary material.

Supplementary file1 (PDF 65 KB)

## Data Availability

Not Applicable.
